# Genetic variation in *SPAG16* regions encoding the WD40 repeats is not associated with reduced sperm motility and axonemal defects in a population of infertile males

**DOI:** 10.1186/1471-2490-12-27

**Published:** 2012-09-10

**Authors:** David R Nagarkatti-Gude, Giulia Collodel, Lori D Hill, Elena Moretti, Michela Geminiani, Zhibing Zhang, Jerome F Strauss

**Affiliations:** 1 Department of Obstetrics and Gynecology, Virginia Commonwealth University, Richmond, 23298, VA, USA; 2 Biochemistry and Molecular Biology, Virginia Commonwealth University, Richmond, 23298, VA, USA; 3 Department of Biomedical Sciences, Applied Biology Section; Interdepartmental Centre for Research and Therapy of Male Infertility, University of Siena, Policlinico Le Scotte, Siena, 53100, Italy; 4 Department of Human and Molecular Genetics, Virginia Commonwealth University, Richmond, USA; 5 MCV Campus, Sanger Hall, 1st Floor, Room 1-071, 1101 East Marshall Street, PO Box 980565, Richmond, 23298, VA, USA

**Keywords:** Sperm ultrastructure, Axoneme, Motile cilia, Male infertility, Central apparatus, Semen analysis

## Abstract

**Background:**

SPAG16 is a critical structural component of motile cilia and flagella. In the eukaryotic unicellular algae *Chlamydomonas*, loss of gene function causes flagellar paralysis and prevents assembly of the “9 + 2” axoneme central pair. In mice, we have previously shown that loss of *Spag16* gene function causes male infertility and severe sperm motility defects. We have also reported that a heterozygous mutation of the human *SPAG16* gene reduces stability of the sperm axonemal central apparatus.

**Methods:**

In the present study, we analyzed DNA samples from 60 infertile male volunteers of Western European (Italian) origin, to search for novel *SPAG16* gene mutations, and to determine whether increased prevalence of *SPAG16* single nucleotide polymorphisms (SNPs) was associated with infertility phenotypes. Semen parameters were evaluated by light microscopy and sperm morphology was comprehensively analyzed by transmission electron microscopy (TEM).

**Results:**

For gene analysis, sequences were generated covering exons encoding the conserved WD40 repeat region of the SPAG16 protein and the flanking splice junctions. No novel mutations were found, and the four SNPs in the assessed gene region were present at expected frequencies. The minor alleles were not associated with any assessed sperm parameter in the sample population.

**Conclusions:**

Analysis of the *SPAG16* regions encoding the conserved WD repeats revealed no evidence for association of mutations or genetic variation with sperm motility and ultrastructural sperm characteristics in a cohort of Italian infertile males.

## Background

Infertility impacts approximately 9% of couples globally, with reports ranging from 3.5% - 16.7% of couples [1], and it is estimated that male factor infertility plays a role in as many as 55% of cases [2]. There are multiple causes of male infertility, including congenital factors and environmental exposures [3-5], as well as gene mutations which cause defects in spermatogenesis and sperm flagellar dysfunction.

The most studied mutations associated with abnormal sperm motility and male infertility cause the primary ciliary dyskinesias (PCD) [6,7]. Genes involved in the structure and function of the “9 + 2” axoneme, the principle scaffold and regulator of motile cilia and flagella, have been extensively studied and characterized in the biflagellate eukaryotic algae *Chlamydomonas*, wherein loss of axonemal components has been shown to cause immotility [8,9]. Most of the mutations identified to date that cause PCD in humans encode proteins that are associated with the axoneme outer doublets. However, relatively little is known about the contribution of mutations or genetic variation in genes encoding the central apparatus of the axoneme to sperm motility defects. It is known that the “9 + 2” axoneme is strongly conserved in overall structure and function [10,11].

We previously reported that mice lacking *Spag6*, which encodes the mammalian orthologue of the *Chlamydomonas* axonemal central apparatus protein PF16, exhibited a phenotype of both male infertility associated with severe sperm motility defects and axonemal structural abnormalities [12], demonstrating that loss of an axonemal central apparatus protein can have dramatic effects in mammals as well.

SPAG16, the mammalian orthologue of the essential *Chlamydomonas* central apparatus protein PF20 [13,14], is also required for male fertility and sperm motility in mice [15]. The *Spag16* gene encodes two major transcripts in mice, which generate SPAG16L, a central apparatus protein, and SPAG16S, a protein localized to the male germ cell nucleus and cytoplasm. Male mice homozygous for a mutation ablating SPAG16L production have a sperm motility defect and are infertile [15]. The axonemes of SPAG16L-deficient mice also show abnormal responses to calcium, indicating that loss of SPAG16L disrupts either the ability to process or the ability to respond to key molecular signaling [16]. When the transcripts encoding SPAG16L and SPAG16S are both disrupted in a transgenic male mouse, there is haploinsufficiency and abnormal spermatogenesis in the chimeric state, implicating SPAG16S in fundamental processes of sperm formation distinct from the structural role of SPAG16L [17]. Importantly, the *Spag16* mutant allele affecting both isoforms was not transmitted to offspring by chimeric males, demonstrating that heterozygous/non-homozygous *Spag16* mutation may cause a pronounced phenotype, and that the two murine SPAG16 isoforms each play different but essential roles.

We have also reported previously that sperm from human subjects heterozygous for a frame shift mutation in S*PAG16* exhibited instability of important central apparatus components SPAG16L, SPAG17, and SPAG6 [18]. While both subjects in this report were fertile, observed biochemical instability of the sperm axoneme suggests that even heterozygous disruption of *SPAG16* has phenotypic consequences that may reduce fecundity. Additionally, a preliminary study of oligozoospermic and oligoasthenozoospermic males has identified 29 previously unreported mutations in *SPAG16* transcripts isolated from ejaculated sperm*,* further suggesting a critical role for *SPAG16* in male fertility [19].

In the present study, 60 males of Western European (Italian) origin volunteered for semen and genomic analyses following diagnosis of male factor infertility. We evaluated the genomic region encoding the highly conserved WD domains of SPAG16 proteins (exons 10–16). WD regions are semi-conserved 20 amino acid sequences giving rise to a β-propeller tertiary structure. These domains are the only known functional domains found in SPAG16 proteins, both isoforms of which contain seven such domains. These β-propeller structures are classically thought to mediate protein-protein interactions (reviewed in [20]), playing crucial roles in macromolecular assembly relevant to a diverse array of cellular tasks, including cell growth and division, intracellular communication, apoptosis, and transcription regulation [21]. Recent reports have also demonstrated that some WD domains interact directly with specific RNA sequences, further expanding the range of potential roles played by members of this class of proteins [22].

To better understand potential links between *SPAG16* gene variation and specific mechanisms of infertility, semen analysis was performed for each subject [23]. In addition, sperm ultrastructure was assessed by transmission electron microscopy (TEM) and described according to previously established standards [24,25].

Based on our previous observations of *Spag16* gene function in mice, and the influence of a heterozygous mutation affecting the WD repeat region in humans on central apparatus stability, we hypothesized that functional SNPs in the human *SPAG16* gene could cause defects in sperm motility and ultrastructure manifest in both the heterozygous and the homozygous states. The present study analyzed genetic variation in a population of infertile males, and their association with parameters that are potentially regulated by *SPAG16*.

## Results

### SPAG16 sequences are highly conserved

**Figure 1:**
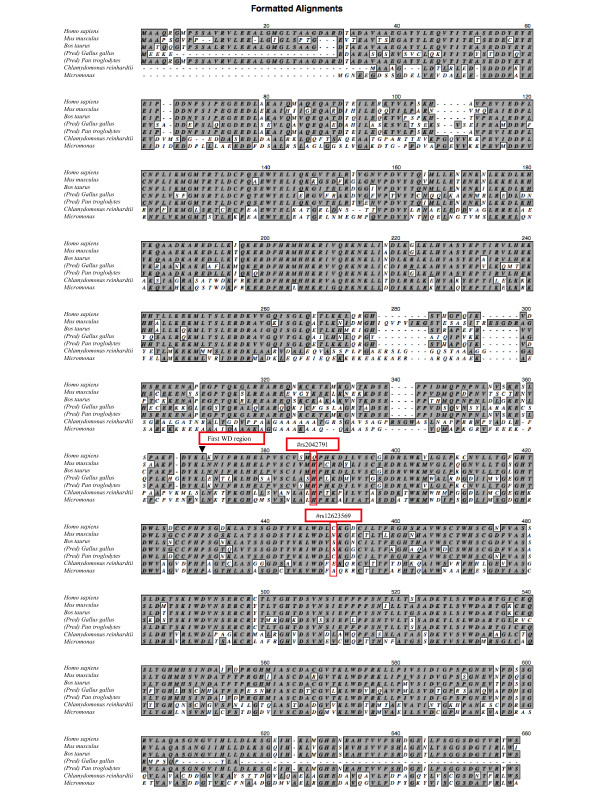
**Alignment of SPAG16 amino acid sequences.** Aligned amino acid sequences are given for full-length proteins in human, mouse, bull, rooster, chimpanzee, *Chlamydomonas*, and *Micromonas.*

In order to establish whether SPAG16 amino acid mutations were likely to result in functional changes, amino acid sequences for several species were aligned to determine regions of conservation within the protein. Alignments are shaded and boxed to indicate identical amino acids relative to the human sequence or conservative substitution (Figure 1). Quantitative assessment demonstrates greater similarity, as expected, between mammalian SPAG16 sequences as compared to the *Chlamydomonas* sequence (Table 1). However, the region most conserved across all species is the C-terminal end, which contains the WD40 repeat domains, the only known conserved domain within these proteins.

**Table 1:** SPAG16 amino acid sequence conservation analysis

	**Homo sapiens**	**Mus musculus**	**Bos taurus**	**(Pred) Gallus gallus**	**(Pred) Pan troglo-dytes**	**Chlamydomonas reinhardtii**	**Micro-monas**	**Similarity Scores (%)**
Homo sapiens	100	74	85.1	49.2	99	35.7	33.6	
Mus musculus	85.1	100	74.6	46.7	74	34.8	34.6	
Bos taurus	92.2	86	100	49.4	85.4	34.9	34.1	
(Predicted) Pan troglodytes	99.5	85.1	92.4	64.5	100	35.7	33.6	
Chlamydomonas reinhardtii	52	51.7	52.9	48.5	52.3	100	52.3	
Micromonas	53.8	52.6	54.4	51.5	54	67.6	100	
Identity Scores (%)								

Quantitative assessment of identity scores (starting from left column) and similarity scores (starting from top row), as calculated by MacVector alignment using default settings.

**Figure 2:**
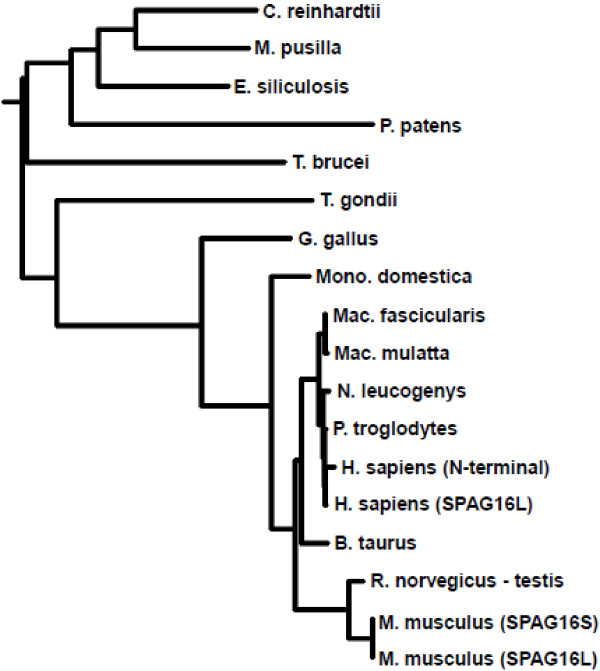
**Sequence analysis of SPAG16 proteins.** Maximum likelihood tree of SPAG16 and SPAG16-orthologous proteins (e.g. PF20). The accession numbers for each entry are listed in the methods section.

While SPAG16 proteins are highly conserved, phylogenetic analysis demonstrates especially strong relationships among mammalian forms, particularly rodents and primates (Figure 2). Only mice have two verified SPAG16 isoforms encoded by two different transcripts: the flagellar form, found in all eukaryotic motile cilia and known in mice as SPAG16L, and a testis-specific SPAG16S, which consists of the WD repeat region. The only known rat SPAG16 is similar in structure to SPAG16S, and was isolated from testis. Furthermore, the nucleotide sequence of the 5^′^-UTR of rat SPAG16 mRNA [GenBank:NM_001134728.1] is similar to that found in mouse SPAG16S mRNA [GenBank:NM_025728.3]. It is reasonable to speculate that a rat SPAG16L protein exists containing both the conserved WD repeats on the C-terminal end and an approximately equally sized N-terminus, consistent with the protein’s structure in all other species.

### Analyzed *SPAG16* SNPs allele frequencies are not altered in the sample population

For one SNP analyzed by Taqman Genotyping Assay, rs10167688, no individuals with the minor allele were detected. This result was further corroborated by exon region-specific sequencing. Heterozygosity for this SNP has been reported only in West African Yoruban populations [26], so its absence in our population was not unexpected. The probe set used to analyze this SNP was confirmed to be effective using a test set of West African female DNA samples obtained in an unrelated study (data not shown).

Two other SNPs were analyzed by TaqMan assay and confirmed by genomic sequencing, which also demonstrated two additional SNPs present within splice junctions. For all four of these SNPs, minor allele frequencies in our patient population did not differ significantly (p > 0.05) from those reported in previous genomic studies of a reference population (Table 2) [26]. Each allele was found to be in Hardy-Weinberg equilibrium. No other alternate alleles corresponding to SNPs identified in previous genomic sequencing studies were found in the sample population. However, the minor allele frequencies for all of putative SNPs not found in the present study population are quite low or unconfirmed in the reference population (Additional file 1: Table S1).

**Table 2:** ***SPAG16*SNP distributions in sample population**

	**rs2042791**	**rs2042792**	**rs12623569**	**rs16851495**
mRNA	A1175C	C [exon 11 3^′^ + 15]G	A1366C	G [exon 14 3^′^ +25] A
Protein	Q361h	-	K425T	-
Homozygous Major allele	19	26	29	43
Heterozygous	33	28	25	17
Homozygous Minor allele	8	6	6	0
Sample population Minor allele frequency	.408	.333	.308	.142
HapMap Minor allele frequency	.39	.39	.30	.08

SNPs rs2042792 and rs16851495 are located in non-coding exon splice regions, while SNPs rs2042791 and rs12623569 are in coding regions of *SPAG16* mRNA, and alter amino acid translation. The nucleic acid and amino acid positions indicated refer, respectively, to mRNA GenBank:NM_024532.*3* and to protein GenBank:NP_078808.3. Expected frequencies refer to the HapMap CEU (European) study [26].

The sequenced region area also covered the following SNPs, for which no minor alleles were found in the sample population: rs10167688, rs115473269, rs61752199, rs2248214, rs28606463, rs114135655, rs113852644, rs117619722, rs6746741, rs71855401, rs12988372, rs12988374, rs80016542. Location and expected frequencies for SNPs not present in the sample population are given in Additional file 1: Table S1.

Major and minor allele representation in the sample population.

Of the four SNPs analyzed, two SNPs were found to be in linkage disequilibrium, rs2042791 and rs2042792 (Table 3).

**Table 3:** Analysis of linkage disequilibrium in exon 11 SNPs

**Haplotype**	**Observed**	**Expected**
CG	0.333	0.136
AG	0	0.197
CC	0.075	0.272
AC	0.592	0.394
	r^2^	.724
	D'	1,000

Linkage equilibrium analysis of two SNPs found in the sample population and present in the genomic proximity: in order, rs2042791 (major A; minor C) and rs2042792 (major C; minor G).

### Analyzed *SPAG16* SNPs are not associated with sperm parameters and alterations of axonemal or periaxonemal ultrastructure, fertility index, and sperm pathologies

In the infertile male population studied, semen analysis revealed severe defects in sperm concentration, motility, and ultrastructure (full data in Additional file 2: Table S2). No single parameter was uniformly perturbed amongst all patients carrying a particular SNP minor allele. Additionally, no mutation was found for which all homo- or heterozygous carriers displayed a similar phenotype beyond deficiencies common to the sample (infertile) population in general. There were nominally significant associations found between specific SNPs and sperm ultrastructural characteristics, but these were not significant after Bonferroni correction for multiple testing. Minor allele carriage at rs16851495 correlated negatively with normal axoneme structures (β = −0.16, nominal p < 0.05; corrected p > 0.1). Minor allele carriage at rs12623569 showed a small, nominally significant, positive correlation with normal fibrous sheath structures (β = 0.09, nominal p < 0.05; corrected p > 0.1) (see Additional file 3: Table S3).

For two SNPs [dbSNP: rs2042791; dbSNP rs2042792] in LD, haplotype was not associated with studied sperm parameters or ultrastructural variation (Additional file 4: Table S4).

### Amino acid-altering *SPAG16* SNPs are predicted to be functionally tolerated

Two of the identified SNPs cause point mutations in SPAG16 proteins. These amino acid substitutions were assessed for predicted effects on protein structure and function. Both amino acid substitutions were predicted to be tolerated using SIFT [27], which analyzes amino acid properties and conserved identities.

**Figure 3:**
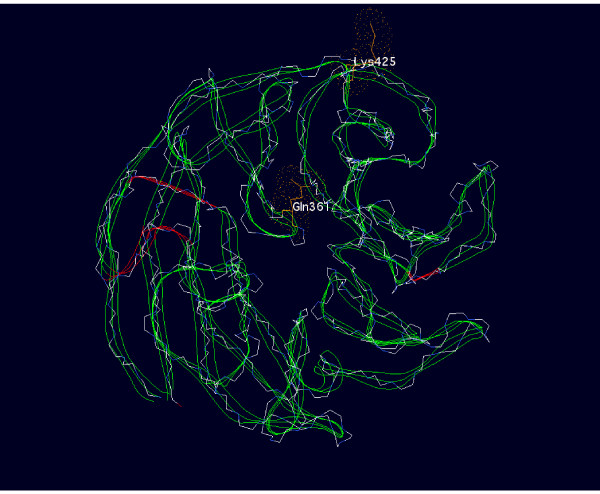
**Predicted approximate 3-D structure of SPAG16.** Predicted 3-D structure of human SPAG16L as determined by overlay on the known structure of WDR5Delta23, the closest related protein with a known crystal structure. Location of SNP-affected amino acids is indicated by shaded sections.

The crystallographic structures of other WD repeat proteins have been determined and these structures can be used to model the structure of the SPAG16 WD repeat region. Using SWISS-MODEL [28], SPAG16L was evaluated for homology with proteins of known structure, and the amino acid sequence was aligned with that of WDR5Delta23, a member of the WD repeat protein family. The locations of the amino acid residues affected by the SNPs evaluated are labeled in the 3-D structure model shown in Figure 3. K425 is predicted to be located on the periphery of the molecule, which is not a known binding site of the structural homologue WDR5Delta23. Q361 extends into the predicted binding pocket.

## Discussion

In the *SPAG16* regions analyzed, both major and minor alleles were present in the study group for four known polymorphisms, and no previously unreported mutations were detected. At rs2042791 [29], the minor allele replacement of an adenosine by a cytosine residue causes the replacement of a glutamine at position 361 [GenBank: NP_078808.3] with a histidine. Previous genotyping studies have reported a minor allele frequency of 0.39 [26] in individuals of Caucasian ancestry for this SNP. Fisher’s exact test demonstrated no significant difference between the measured minor allele frequency of 0.408 in our sample population and that established in control populations (p > 0.05). While the present study lacks sufficient power to detect small effects of these alleles on variation in observed parameters of sperm characterization, among patients carrying this SNP no single factor was uniformly deficient within the distribution of sub-normal semen analysis of the study participants. Full genotype and semen analysis results are included for reference (Additional file 2: Table S2).

The lack of association between this SNP and sperm dysfunction is not surprising, given that the glutamine replacement by histidine is predicted to be tolerated in the protein structure [27]. Further, the amino acid is not conserved in mammals – *Mus musculus* [GenBank:AAI20669.1, NP_080004.1, NP_083436.2] and *Rattus norvegicus* [GenBank:NP_001128200.1, AAI58603.1] reference sequences for the orthologous proteins in fact report a histidine at this position, consistent with the human minor allele. The same amino acid replacement is predicted in *Pan troglodytes* [GenBank:XP_001148592.1, XP_526016.2, XP_001148393.1]. These data are consistent with the hypothesis that structural rather than sequence homology is essential to maintenance of axoneme function.

The rs2042792 minor allele was not found at a significantly different frequency than in the reference population (p > 0.05), and did not correlate with assessed sperm phenotypes.

Rs2042791 exists in LD with rs2042792 in our population, but no phenotypic consequences were associated with any of the observed haplotypes.

Minor allele replacement of an adenosine with a cytosine in rs12623569 causes an amino acid replacement at position 425 [GenBank:NP_078808.3], with a threonine taking the place of a lysine. Previous genotyping studies have shown a minor allele frequency for this SNP in individuals of Caucasian ancestry of 0.30 [26]. The present sample population exhibited a minor allele frequency of 0.333, which was not significantly different than that in control populations (p > 0.05). While the lysine at position 425 encoded by the major allele is present in other mammals (*Mus musculus* – GenBank:NP_080004.1, NP_083436.2; *Rattus norvegicus* – GenBank:NP_001128200.1, AAI58603.1), replacement by threonine is predicted to be tolerated by the protein structure. Interestingly, minor allele carriage was found to be nominally associated with a higher percentage of normal accessory fibers of the axoneme. Although the statistical significance of this association was lost upon correction for multiple testing, further studies on a larger study population may be warranted to identify contributions of the SNP to sperm flagellar structure.

At rs16851495, minor allele replacement of guanine with an adenine residue does not affect protein translation directly, as the position lies outside the translated exon region. However, minor allele carriage was nominally associated with the reduced presence of normal axoneme structure in the study population. The statistical significance of this association did not withstand correction for multiple testing. However, the limited power of our study may have contributed to the absence of a robust association.

For all SNPs discussed, both heterozygous and homozygous individuals were present in the sample population, and statistical analysis demonstrated no difference in genotype frequency between the sample and control populations (p > 0.05). While the study did not have sufficient power to analyze possible contributions to complex traits, the results negate the hypothesis that amino acid modification at the tested positions may result in a loss of protein function that would mimic the severe phenotypes observed in transgenic mouse studies.

Our results suggest that non-synonymous amino acid substitutions at residues 361 and 425 in the SPAG16 protein are not sufficient to explain a reduction of sperm motility and fertility index, the presence of axonemal/periaxonemal alterations, or an increased percentage of sperm pathologies in the assessed patient population. Based on the strong homology between members of the WD protein family, it is tempting to suspect that these resides may be non-essential in other WD repeat proteins as well.

The profound defects observed in studies of *Spag16* gene effects in other species suggest that a functional mutation would significantly perturb sperm function, and would be observed even in a small sample population. Our observations do not, however, preclude the possibility that alternative variations in the *SPAG16* gene cause an increase in sperm ultrastructural alterations, and a reduction in sperm motility. Indeed, our previous studies suggest that more significant SPAG16 mutations, such as a frame shift, can reduce sperm central apparatus stability. Further studies of *SPAG16* gene variation are warranted to offer a comprehensive understanding of the gene’s contributions to male fertility. The sensitivity of the present study to detect variation with the sample is limited by the lack of similarly extensive sperm analysis data, in particular TEM ultrastructural analysis, from a control population. Future development of this area of research should include control samples from men with known fertility and/or sperm count, motility, and structure in the normal range.

Future studies in mammals are necessary as well to explore the nature of *Spag16* gene evolution, and the possibility that male germ cell-specific isoforms exist in multiple species. The only identified rat SPAG16 protein is similar to SPAG16S in size, and is derived from mRNA similar to murine *SPAG16S*, with a 5^′^UTR from the region upstream of the first exon suggestive of independent transcription, rather than splice variation. A putative *SPAG16S* promoter and 5′UTR have been identified in humans as well [GenBank: EF591776.1], raising the possibility that SPAG16S maybe be common to mammalian male germ cells. We have recently shown that murine SPAG16S is enriched within nuclear speckles of male germ cells [30]. This unique localization, combined with phenotypic results from various *Spag16* transgenic mice, strongly suggests a unique processing role for the SPAG16S protein, and thus a distinct role for the *Spag16* gene apart from its structural role in the axoneme.

## Conclusions

In a sample population of 60 infertile males of Western European origin, mutations in *SPAG16* were not significantly associated with a single phenotype of sperm alteration. The findings suggest that mutations and or genetic variation in the *SPAG16* regions encoding the protein’s WD repeats are not likely to be major causes of sperm motility and sperm flagellar defects.

## Methods

### DNA sample acquisition

DNA used in the present study was isolated from peripheral blood lymphocytes submitted by 60 male volunteers of Western European (Italian) ancestry diagnosed clinically as sub- or in- fertile. Inclusion criteria for patients were: sperm progressive motility ≤ 10%, normal lymphocyte karyotype, normal hormone levels, absence of anatomical pathologies, genitourinary infections. Patients with known ultrastructural sperm defects of possible genetic origin (dysplasia of the fibrous sheath, cilia immotile syndrome, round headed sperm, etc.) were also excluded.

## Ethics

Research was carried out in full compliance with the Helsinki Declaration of ethical principles for medical research involving human subjects. All patients signed a declaration of informed consent to participate in the research. The work was approved of and performed in full accordance with policies governing human subject research at the by the Ethics Committee for the University of Siena (CEL-AOUS) for specimens collected by non-invasive methods for clinical analysis, and the Virginia Commonwealth University Institutional Review Board.

### Genotyping

Allelic genotyping was performed using Applied Biosystems TaqMan SNP Genotyping Assays according to manufacturer’s instructions. Specific allelic discrimination probes and primer sets were ordered for the following 3 SNPs: dbSNP: rs2042791, rs12623569, rs10167688. Results were compared to allele frequency detected in NCBI and HapMap studies [26,29].

Genotype was confirmed by direct sequencing. Briefly, exon plus flanking regions were amplified by PCR and sequenced using tested primers. Primer suitability for sequencing was tested by comparing results to sample PCR products digested and amplified in a Topo TA Vector (Invitrogen).

PCR and Sequencing Primers

Ex10 – Forward: 5^′^ - TTCATGTAAATTCTGGGCAAA - 3^′^; Reverse: 5^′^ - GCAAACCATTTCAACCATGA - 3^′^

Ex11 – Forward: 5^′^ - TGGGGCCAGTACTCTCAAAA - 3^′^; Reverse: 5^′^ - TTCAGTGCAGGGTGTGTTGT - 3^′^

Ex12 – Forward: 5^′^ - GCAATTCAAGTTAGCAATTGTG - 3^′^; Reverse: 5^′^ - CCTGGGGTAGCATCAAGG - 3^′^

Ex13 – Forward: 5^′^ - TTATTTCATGCCTCAGTTCCTT - 3^′^; Reverse: 5^′^ - GCCCTTGCACAATCACTTTT - 3^′^

Ex14 – Forward: 5^′^ - GGGAGGAGGGGCTAAAAATTA - 3^′^; Reverse: 5^′^ - CCTAAAGTTGTTCTTCTCACCTCA - 3^′^

Ex15 – Forward: 5^′^ - AGAGGAATGTAATCTTATGGCTGT - 3^′^; Reverse: 5^′^ - TTCATATGACATGCTATACGTAATGA - 3^′^

Ex16 – Forward: 5′ - CTGACCCCTAACACAGAATGA - 3^′^; Reverse: 5^′^ - CCAGGTTTTCCTGCAGTTT - 3^′^.

### Statistical analysis

Fisher’s exact tests implemented in the open-source *R* software package were used to test for differences between allele frequencies observed in the sample population and those reported in HapMap [31]. Hardy-Weinberg equilibrium tests and inter-SNP linkage disequilibrium calculations were performed using PLINK [31]. Haplotypes were assigned to subject based on the most likely phase reconstructed haplotype generated by the expectation-maximization algorithm implemented in PLINK. Haplotypes were then imported to *R* and an additive term for the haplotype of interested was coded as 0, 1, or 2 based on copy number present. Single SNP and haplotype associations with all phenotypes were carried out using multiple logistic regression in *R*. Phenotypes measured as percentages were transformed with arcsine of the square root of the percentage to stabilize the differences in variance between samples. The Fertility Index is a standard measure ranging from 0 to 100+. All values greater than 100 were coded as 100. To achieve a more normal distribution and minimize residual errors, sperm/mL was analyzed using a log transformation. A Bonferroni *p* value was calculated to correct for multiple testing error.

### Sperm analysis

Semen samples of patients were collected by masturbation after 4 days of sexual abstinence and examined after liquefaction for 30 min at 37°C. Volume, pH, concentration (sperm x 10^6^/mL) and total motility (a, rapid + b, slow, + *in situ*) were evaluated according to World Health Organization guidelines [23]. For electron microscopy, sperm samples were prepared as previously described [24,25].

In each sample the percentage of normal axonemal pattern, of coiled axonemes, of well-assembled fibrous sheaths and accessory fibers were estimated by TEM. Moreover, all TEM data were elaborated using the statistical mathematical formula by Baccetti et al. [25], based on the Bayesian method, which calculates the number of spermatozoa probably free of structural defects (fertility index, FI) and the percentages of three main phenotypic sperm pathologies: immaturity, necrosis and apoptosis [24].

### Molecular structure prediction

Using SWISS-MODEL [28], mouse SPAG16L was evaluated for homology with proteins of known structure, and the results mapped on the 3-D structure of the most closely related homologue.

The molecular analysis tool SIFT [27] was used to predict whether the analyzed amino acid substitutions would be tolerated, based on sequence homology and amino acid physical properties.

### Amino acid alignment

Alignment analysis, including scoring of conservative versus semi-conservative or radical amino acid substitutions, was performed using recommended default settings in MacVector 11.6 (MacVector. Inc.; Cary, NC) for the following isoforms:

*Homo sapiens* – GenBank:NP_078808.3

*Mus musculus* – GenBank:NP_083436.2

*Bos taurus* - GenBank:DAA32462.1

*Gallus gallus* - GenBank:XP_421865.2 (predicted)

*Pan troglodytes -* GenBank: XP_001148592.1 (predicted)

*Chlamydomonas reinhardtii* - GenBank:AAB41727.1

*Micromonas sp. RCC299* - GenBank:ACO61299

### Maximum likelihood tree

Amino acids sequences were aligned by Clustal W [32] using default settings, hosted by GenomeNet at the Kyoto University Bioinformatics Center [33]. Aligned sequences were analyzed for maximum likelihood tree and formatted for figure output using programs in the Phylip 3.69 software package [34]. Isoforms used were as follows:

*C. reinhardtii* – GenBank:AAB41727.1

*M. pusilla* CCMP1545 – GenBank:EEH57835.1

*E. siliculosis* – GenBank:CBN79843.1

*P. patens* – GenBank:EDQ58898.1

*T. brucei* – GenBank:AAC83819.1

*T. gondii* ME49 – GenBank:EEA99732.1

(Rooster) *G. gallus* – GenBank:XP_421865.3 (Predicted)

(Opossum) *M. domestica* - GenBank:XP_003341903.1 (Predicted)

(Macaque f.) *M. fascicularis* – GenBank:BAE01109.1

(Rhesus macaque) *M. mulatta –* GenBank:XP_001082826.2 (Predicted)

(Gibbon) *N. leucogenys* - GenBank:XP_003254083.1 (Predicted)

(Chimpanzee) *P. troglodytes* - GenBank:XP_001148592.1 (Predicted)

(Human) *H. sapiens* N-terminal variant – GenBank:NP_001020607.1

(Human) *H. sapiens* SPAG16L – GenBank:NP_078808.3

(Bull) *B. taurus* - GenBank:DAA32462.1

(Rat) *R. norvegicus* (discovered in testis; SPAG16S homologue) – GenBank:AAI58603.1

(Mouse) *M. musculus* SPAG16S – GenBank:NP_080004.1

(Mouse) *M. musculus* SPAG16L – GenBank:NP_083436.2.

## Abbreviations

SNPs = Single nucleotide polymorphisms; PCD = Primary ciliary dyskinesias; TEM = Transmission electron microscopy; UTR = Untranslated region; LD = Linkage disequilibrium.

## Competing interests

The authors declare that they have no competing interests.

## Authors’ contributions

DRN-G designed and carried out the genetic studies and drafted the manuscript. GC, EM, and MG acquired participant samples and carried out sperm analysis. LDH carried out statistical analysis and participated in genetic studies. ZZ and JFS conceived of the study and participated in its design and coordination. All authors read and approved the final manuscript.

## Pre-publication history

The pre-publication history for this paper can be accessed here:

http://www.biomedcentral.com/1471-2490/12/27/prepub

## Supplementary Material

Additional file 1 **Table S1.** Minor allele frequencies of all SNPs in the tested SPAG16 region. Expected minor allele frequencies refer to HapMap CEU European population [26] where available. Unknown = no data available for suitable reference population; * = Pilot 1 HapMap CEU panel, 60 individuals. Click here for file

Additional file 2 **Table S2.** Tests for association between all SNPs in sample population and sperm phenotype. Association tests for each SNP found in the sample population and studied indicators of sperm structure and function. * = data assessed using arcsine of the square root of the phenotype; ^ = data assessed using log transformation of the phenotype; c = Bonferroni correction results in p > 0.1. Click here for file
