# Supplementary material for: Genetic variation in SPAG16 regions encoding the WD40 repeats is not associated with reduced sperm motility and axonemal defects in a population of infertile males
Source: BMC Urol. 2012 Sep 10;12:27. doi: 10.1186/1471-2490-12-27 (PMC3487941; doi:10.1186/1471-2490-12-27)
Supplement: Additional file 1 — Table S1. Minor allele frequencies of all SNPs in the tested SPAG16 region. Expected minor allele frequencies refer to HapMap CEU European population [26] where available. Unknown = no data available for suitable reference population; * = Pilot 1 HapMap CEU panel, 60 individuals. [file 1471-2490-12-27-S1.pdf]

Exon	SNP	Sample minor allele freq.	Expected minor allele freq.	SNP	Sample minor allele freq.	Expected minor allele freq.	SNP	Sample minor allele freq.	Expected minor allele freq.
**10**	**rs10167688** [C1062A; P324T]	0.00	0.00	**rs115473269** [T11116A ; Y342N]	0.00	unknown	**rs61752199** [G11159A; S356N]	0.00	.033*
**11**	**rs2248214** [5' -3 G/T]	0	unknown	**rs2042791** [A1175C ; Q361H]	.408	0.39	**rs2042792** [3' +15 C/G]	.333	0.39
**12**	**rs114135655** [5' -26 G/C]	0	unknown	**rs28606463** [5' -25 C/T]	0.00	unknown	**rs12623569** [A1366C; K425T]	.308	0.28
**13**	no known/detected SNPs								
**14**	**rs113852644** [A1663G; N524S]	0.00	unknown	**rs117619722** [3' +12 C/T]	0.00	unknown	**rs16851495** [3' +25 G/A]	.142	.08
**15**	**rs71855401** [5' -31 2bp delet]	0.00	unknown	**rs6746741** [T1715C; no mutation]	0	0			
**16**	**rs12988372** [C1946T; no mutation]	0.00	unknown	**rs12988374** [C1951T; S620F]	0.00	unknown	**rs80016542** [G1975A; R628Q]	0.00	unknown
