# Supplementary material for: Genetic variation in SPAG16 regions encoding the WD40 repeats is not associated with reduced sperm motility and axonemal defects in a population of infertile males
Source: BMC Urol. 2012 Sep 10;12:27. doi: 10.1186/1471-2490-12-27 (PMC3487941; doi:10.1186/1471-2490-12-27)
Supplement: Additional file 2 — Table S2. Tests for association between all SNPs in sample population and sperm phenotype. Association tests for each SNP found in the sample population and studied indicators of sperm structure and function. * = data assessed using arcsine of the square root of the phenotype; ^ = data assessed using log transformation of the phenotype; c = Bonferroni correction results in p > 0.1. [file 1471-2490-12-27-S2.pdf]

| SNP | MAF | normal acc. fibers* | | coiled axonemes* | | normal 9+2 axoneme* | | normal fibrous sheath* | | % immaturity* | | fertility index (FI) | | *in situ* motility | | motility 'a' | | motility 'b' | |
|---|---|---|---|---|---|---|---|---|---|---|---|---|---|---|---|---|---|---|---|
| | | **β (SE)** | **p** | **β (SE)** | **p** | **β (SE)** | **p** | **β (SE)** | **p** | **β (SE)** | **p** | **β (SE)** | **p** | **β (SE)** | **p** | **β (SE)** | **p** | **β (SE)** | **p** |
| rs2042791 | 0.408 | 0.03 (0.04) | 0.499 | 0.00 (0.04) | 0.964 | 0.00 (0.06) | 0.998 | 0.01 (0.04) | 0.879 | -0.02 (0.03) | 0.442 | 2.10 (12.56) | 0.868 | -0.48 (0.83) | 0.563 | -0.14 (0.50) | 0.785 | 0.30 (0.59) | 0.615 |
| rs2042792 | 0.333 | 0.05 (0.04) | 0.207 | -0.01 (0.04) | 0.774 | 0.04 (0.06) | 0.479 | 0.01 (0.04) | 0.835 | -0.01 (0.03) | 0.829 | -16.83 (13.14) | 0.209 | 0.05 (0.84) | 0.955 | 0.00 (0.50) | 0.994 | 0.25 (0.59) | 0.673 |
| rs12623569 | 0.282 | 0.01 (0.04) | 0.702 | 0.02 (0.03) | 0.601 | 0.00 (0.05) | 0.978 | 0.09 (0.04) | **0.014**[c] | 0.04 (0.03) | 0.1 | 20.80 (10.72) | 0.06 | 1.13 (0.74) | 0.132 | 0.31 (0.45) | 0.5 | -0.65 (0.53) | 0.221 |
| rs16851495 | 0.142 | -0.12 (0.06) | **0.041**[c] | 0.01 (0.05) | 0.873 | -0.16 (0.08) | **0.039**[c] | -0.03 (0.06) | 0.674 | -0.03 (0.04) | 0.515 | -10.07 (16.19) | 0.538 | -0.90 (1.18) | 0.453 | -0.71 (0.70) | 0.32 | 0.10 (0.85) | 0.905 |

| SNP | MAF | motility 'a+b' | | total motility | | necrosis | | % necrosis* | | sperm/mL^ | | volume/mL | | total sperm | | apoptosis | | % apoptosis* | |
|---|---|---|---|---|---|---|---|---|---|---|---|---|---|---|---|---|---|---|---|
| | | **β (SE)** | **p** | **β (SE)** | **p** | **β (SE)** | **p** | **β (SE)** | **p** | **β (SE)** | **p** | **β (SE)** | **p** | **β (SE)** | **p** | **β (SE)** | **p** | **β (SE)** | **p** |
| rs2042791 | 0.408 | 0.16 (0.76) | 0.831 | -0.32 (1.25) | 0.798 | -0.78 (5.1) | 0.878 | 0.00 (0.06) | 0.991 | -0.13 (0.34) | 0.715 | -0.21 (0.47) | 0.66 | 4.45 (20.26) | 0.827 | -1.94 (2.28) | 0.399 | -0.04 (0.03) | 0.292 |
| rs2042792 | 0.333 | 0.26 (0.76) | 0.739 | 0.30 (1.25) | 0.81 | -6.21 (5.04) | 0.224 | -0.06 (0.06) | 0.303 | -0.55 (0.35) | 0.128 | 0.04 (0.47) | 0.939 | 18.60 (20.25 | 0.363 | -1.75 (2.30) | 0.45 | -0.03 (0.03) | 0.305 |
| rs12623569 | 0.282 | -0.35 (0.69) | 0.616 | 0.78 (1.13) | 0.49 | -2.73 (4.60) | 0.556 | -0.04 (0.05) | 0.471 | 0.44 (0.32) | 0.178 | -0.25 (0.42) | 0.55 | 12.83 (18.33 | 0.487 | -1.93 (2.07) | 0.356 | -0.03 (0.03) | 0.361 |
| rs16851495 | 0.142 | -0.61 (1.08) | 0.579 | -1.50 (1.77) | 0.401 | 11.55 (7.10) | 0.11 | 0.14 (0.08) | 0.084 | 0.46 (0.53) | 0.382 | 0.24 (0.70) | 0.723 | 47.74 (29.11 | 0.107 | 1.68 (3.28) | 0.611 | 0.02 (0.05) | 0.637 |
